# Revision arthrodesis of the ankle: Posterior inlay bone grafting in 11 patients

**DOI:** 10.3109/17453670902884791

**Published:** 2009-04-01

**Authors:** Fleur V Verhulst, Bart A Swierstra

**Affiliations:** Department of Orthopaedic Surgery, Sint MaartenskliniekNijmegenThe Netherlands

## Introduction

We present a posterior inlay bone grafting technique that was originally described for primary ankle arthrodesis (White 1974, Swärd et al. 1992) but which also appears to be useful for revision of ankle pseudoarthrosis.

## Technique

**Figure 1.**
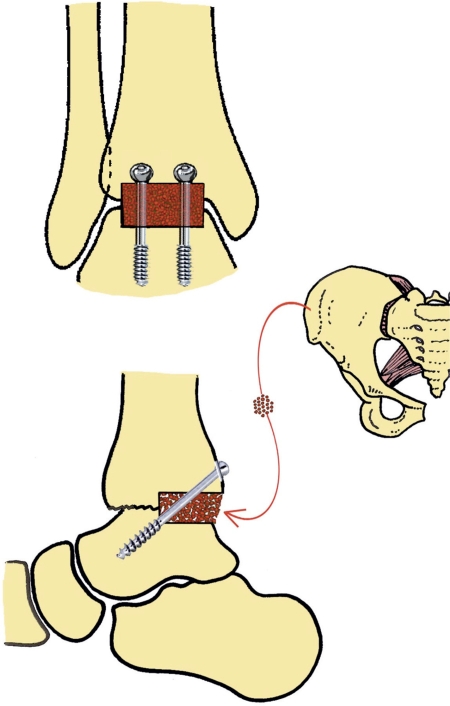
Technique of revision ankle arthrodesis with posterior inlay bone grafting.

The patient is placed in a prone position with a tourniquet around the upper leg. A posterior longitudinal incision is made medially to the Achilles tendon, which is exposed and divided by a Z-plasty. The posterior compartment is opened to provide access to the distal tibia and the talocrural pseudoarthrosis. When there is macroscopic instability, existing hardware is removed and 2 cannulated screws are put in from the posterior tibial cortex into the talar body and neck. Their position is checked with an image intensifier. If existing hardware cannot be removed without major exposure (e.g. when original screws are broken) or if skeletal deformity precludes the use of 2 posterior screws, an external fixator is used with 2 pins through the calcaneus and 2 pins through the tibia, connected by a bilateral rod. A 3-cm × 2-cm wide and 2-cm deep slot is cut into the talocrural area, including removal of the pseudoarthrosis. An ample amount of cancellous bone chips from the posterior iliac crest is packed into the slot (Figure 1).

The postoperative regimen starts with 6 weeks without any weight bearing in cases of internal fixation in a below-knee plaster cast and in cases of external fixation without external support. Thereafter, weight bearing in a plaster cast and removal of the external fixation depends on radiographic judgment of bone healing.

## Patients

**Figure 2.**
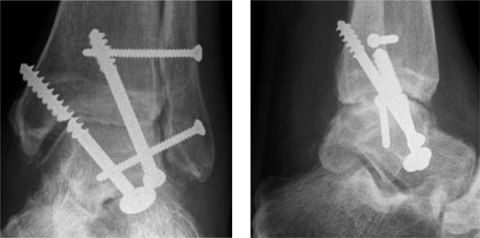
Patient 6. A. Pseudoarthrosis with screw breakage 4 months after the first operation. B. Fusion 11 weeks after removal of broken screws, refixation with posterior screws, and posterior inlay bone grafting.

Between 2004 and 2007, 11 patients with radiographically confirmed pseudoarthrosis of the ankle were operated on with this technique (Table 1). Their mean age at operation was 52 (29–80) years. The average time from the last arthrodesis attempt until revision arthrodesis was 8 (2–19) months. Primary diagnosis included: osteoarthritis (8), rheumatoid arthritis (1), Charcot Marie tooth disease (1), and polio paralysis (1). In 4 patients, an external fixator was used. Fusion occurred in 10 of the 11 patients (Figure 2). The mean time to full, unprotected weight bearing was 14 (6–22) weeks.

**Figure:**
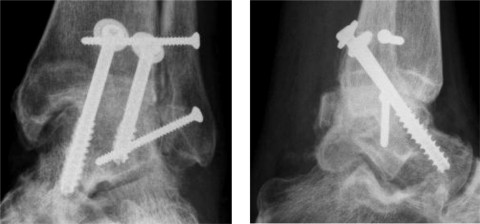
B. Fusion 11 weeks after removal of broken screws, refixation with posterior screws, and posterior inlay bone grafting.

**Table 1.** Clinical data

Case	M/F	Age	Months since last surgery	Number of previous attempts at arthrodesis	Previous fixation method **^a^**	New fixation method **^b^**	Weeks to full, unprotected weight bearing	Complications **^c^**	Comments
1	M	48	6	3	IM rod, screws	None	13	–	–
2	F	29	6	2	IM rod, screws	None	6	–	–
3	M	48	10	1	Screws	None	12	–	–
4	M	57	5	2	Screws	External fixator	–	A	Pseudoarthrosis
5	M	58	19	1	Screws	None	9	B	–
6	M	39	4	1	Screws	Posterior screws	11	–	–
7	M	53	8	1	IM rod, screws	External fixator	16	C	–
8	M	59	2	2	Screws, Steinmann pins	External fixator	22	–	–
9	M	80	11	1	Screws	Posterior screws	15	–	–
10	M	57	10	1	Screws	Posterior screws	17	–	–
11	M	46	6	1	Screws	External fixator	17	C	–

**^a^** IM: Intramedullary

**^b^** None: previous osteosynthesis material not removed, only bone grafting performed.

**^c^** Complications: A: Slow wound healing; B: Late infection (4 weeks) iliac crest wound; C: Pin tract infection

Complications occurred in 4 patients and included: 1 slow wound healing, 1 low-grade late infection (after 4 weeks) of the iliac crest wound, and 2 pin tract infections of external fixators. The infections healed by conservative means and did not compromise the outcome.

## Discussion

Pseudoarthrosis occurs in 0–40% of all ankle fusions (Saltzman 2008). Different procedures have been tried for revision arthrodesis. Most series have been small, like ours, and the results have been similar: fusion occurs often but not always, and complications are common (Table 2). Bone grafts are widely used but Kitaoka et al. (1992) and Anderson et al. (1997) could not find a correlation between bone grafting and union rate, probably due to the small number of patients. The best way to approach the ankle (anteriorly, posteriorly, or laterally) has been discussed (Hayes and Nadkarni 1996, Klaue and Bursic 2005, Hammit et al. 2006). The advantages of our posterior approach are the avoidance of old scars and the danger of disturbed wound healing (Kile et al. 1994, Hammit et al. 2006), the ample amount of healthy tissue covering the arthrodesis site, and minimal disturbance of local osseous vascularization. Our technique is, however, less suitable for correction of coexisting deformities.

**Table 2.** Results of revision ankle arthrodesis

Author	Approach	Fixation method **^a^**	n	Bone graft	Fusions	Consolidation mean (range), weeks	Complications **^b^**	Comments **^c^**
Kirkpatrick	Antero-lateral	IF	11	11	9	20 (?)	5	
Kitaoka (1992)	Medial or lateral	EF	26	18	20	?	7	
Eingartner	Previous	EF	16	6	15	12 (11–16)	6	
Levine	Previous	EF (5) or IF (18)	23	14	21	14 (6–48)	8	A
Anderson **^d^**	Lateral	IF	13	13	8	24 (8–68)	13	B
Midis	Lateral	EF	10	10	10	13 (9–20)	2	
Cheng **^d^**	Anterior	IF	10	none	9	19 (10–40)	No data	C
Katsenis **^d^**	?	EF	10	9	10	31 (18–62)	See below**^b^**	D
Our study	Posterior	EF (4) or IF (7)	11	11	10	14 (6–22)	4	

**^a^** IF: internal fixation; EF: external fixation.

**^b^** 61 complications in 21 nonunion and malunion patients.

**^c^** Comments: A: Additional hindfoot arthrodesis in 9 patients; B: 3 amputations; C: 2 delayed union; D: Additional leg lengthening in 6 patients

**^d^** These authors included nonunion and malunion patients in their studies; in this overview however, only the nonunion patients are considered.

Several authors have used clinical rating criteria to assess function, pain, and alignment after revision arthrodesis (Midis an Conti 2002, Cheng et al. 2003, Levine et al. 1997, Anderson et al. 1997). We chose not to make use of any of these rating criteria because they do not specifically assess the results of the technique of revision arthrodesis per se, but rather reflect the underlying condition.

This technique of ankle arthrodesis with bone grafting by a posterior approach has not been used before as a revision technique after pseudoarthrosis. Fusion time and complications compared favorably with other techniques.
